# Problematic drinking in the old and its association with muscle mass and muscle function in type II diabetes

**DOI:** 10.1038/s41598-019-47787-0

**Published:** 2019-08-19

**Authors:** Nikolaus Buchmann, Dominik Spira, Maximilian König, Kristina Norman, Ilja Demuth, Elisabeth Steinhagen-Thiessen

**Affiliations:** 1 Charité – Universitätsmedizin Berlin, corporate member of Freie Universität Berlin, Humboldt-Universität zu Berlin, and Berlin Institute of Health, Lipid Clinic at the Interdisciplinary Metabolism Center, Berlin, Germany; 2 Department of Internal Medicine B, University Medicine Greifswald, Greifswald, Germany; 3 German Institute of Human Nutrition, Department of Nutrition and Gerontology, Potsdam-Rehbruecke (DIfE), Nuthetal, Germany; 4 Charite - Universitätsmedizin Berlin, Forschungsgruppe Geriatrie am EGZB, Berlin, Germany; 5 Charité - Universitätsmedizin Berlin, BCRT - Berlin Institute of Health Center for Regenerative Therapies, Berlin, Germany

**Keywords:** Endocrinology, Health care

## Abstract

Problematic drinking behavior is common in the old and negative consequences of hypoglycemic episodes in type 2 diabetes (T2D) as a result of alcohol consumption have been described previously. Although, associations between such hypoglycemic episodes with reduced muscle mass are discussed, it is uncertain if problematic drinking behavior drives decline of muscle mass and/or muscle function. In the current study, we analyzed data of the Berlin Aging Study II (BASE-II) to examine the association of problematic drinking behavior with muscle mass and grip strength in T2D. Cross-sectional data of 1451 old BASE-II participants (51.6% women; 60–84 years old) were analyzed. Problematic drinking behavior was assessed using the Alcohol Use Identification Test (AUDIT). Muscle mass was measured using dual energy X-ray absorptiometry (DXA), grip strength using a Smedley dynamometer. Adjusted regression models were calculated to assess the association of problematic drinking with muscle mass and grip strength. Problematic drinking was evident in 11.2% of BASE-II participants and in 12.5% of BASE-II participants diabetes was evident. In the fully adjusted model (adjusted for age, trunk fat mass, HbA1c, antidiabetic medication, TSH, CRP, testosterone, physical inactivity, depression (GDS-score), morbidities, smoking status and total energy intake/day, we found a statistically significant association between problematic drinking and muscle mass (β-3.7, SE: 1.3, R^2^ 0.481, partial eta square 0.166, observed power 0.816, p-value 0.005) and grip strength (β-8.1, SE: 3.3, R^2^ 0.222, partial eta square 0.134, observed power 0.670, p-value 0.018) in old diabetic men. These associations were not evident in women and subjects without T2D. Problematic drinking behavior was associated with lower muscle mass and grip strength in old men with diabetes. This topic should be addressed in these subjects as they could be at increased risk for early functional decline, sarcopenia or frailty.

## Introduction

Alcohol consumption and problematic drinking behavior can frequently be observed in all age groups^1–3^. Although the prevalence of problematic drinking behavior decreases in advanced age, additional implications of alcohol consumption have to be taken into account in older people. With an increasing number of morbidities and medications, old subjects face a higher risk for negative consequences of alcohol consumption like liver damage, falls, fractures, malnutrition or cognitive decline^4–6^. In addition, metabolism and distribution of alcohol in the body is altered in the old^7^. Body composition changes with aging and an increased proportion of body fat and reduced intracellular water is recognized in the old. Moreover, decreased activity of alcohol dehydrogenase and increase of organ sensitivity for alcohol in the old put these subjects at increased risk for alcohol toxicity and thus negative consequences of alcohol^8,9^. Notably, loss of muscle mass (i.e. as a consequence of alcohol-induced autophagy or due to impaired skeletal muscle protein synthesis) and elevation of creatine kinase (CK) have been observed as a consequence of alcohol consumption^10,11^.

This is particularly emerging in subjects with type II diabetes (T2D). In addition to age-related conditions, hypoglycemic states are particularly common in T2D and have also been associated with elevation of creatine kinase (CK), alterations in isoenzyme distribution of CK and serum enzyme activity of liver enzymes in an animal model^12^. Muscle is the main tissue involved in glucose metabolism and hypoglycemic conditions due to alcohol consumption in T2D might result in accelerated muscle breakdown. Therefore, an association between muscle deterioration and alcohol consumption in T2D seems plausible. Surprisingly, a recent meta-analysis suggested that alcohol consumption is not associated with the development of sarcopenia^13^.

The aim of the current study on 1451 old participants of the Berlin Aging Study II (BASE-II) was to analyze the association of problematic drinking behavior with muscle mass (appendicular lean mass; ALM) and muscle function (grip strength). We hypothesized that reduced muscle mass and muscle function are especially pronounced in diabetics with problematic drinking behavior. As muscle mass and muscle function are affected by various parameters, we adjusted for a number of potential confounders such as medication, age, trunk fat mass, endocrine and inflammatory parameters (TSH, Testosterone, CRP), physical inactivity, depression, morbidities, smoking status and dietary habits. Problematic drinking behavior was assessed using the AUDIT (Alcohol Use Disorders Identification Test) questionnaire and problematic drinking behavior was defined as AUDIT-Score > 5 in women or >8 in men^14,15^. Thus, the focus of the analysis was not on the assessment of type or amount of alcohol but on unfavorable drinking habits and drinking behavior.

## Methods

### Study population

The study population investigated here consisted of 1451 older participants of the Berlin Aging Study-II (BASE-II, 50.8% women; 68 ± 4 years old). We analyzed cross-sectional data of BASE-II, a prospective epidemiological study, which previously has been described in detail^16,17^. The medical examination in BASE-II consisted of a two-day protocol including a comprehensive anamnesis performed by a physician and a wide array of laboratory and functional tests. The study was approved by the ethics committee of Charité – Universitätsmedizin Berlin (project number: EA2/029/09). All subjects gave written informed consent to participate in the study. The study was conducted according to the declaration of Helsinki.

### Type II diabetes (T2D) and drinking behavior

T2D was defined according to the ESC-guidelines (European Society of Cardiology) as: (i) anamnestic information of T2D or diabetes-specific medication, (ii) fasting glucose (after >8 hours fasting period) > 126 mg/dl, (iii) post-load glucose > 200 mg/dl (120 minutes after glucose load; when available), or iv) HbA1c > 6.5%^18^. Problematic drinking behavior was assessed using the AUDIT (Alcohol Use Disorders Identification Test) questionnaire. The questionnaire includes the following questions: 1. How often do you have a drink containing alcohol? 2. How many standard drinks containing alcohol do you have on a typical day when drinking? 3. How often do you have six or more drinks on one occasion? 4. During the past year, how often have you found that you were not able to stop drinking once you had started? 5. During the past year, how often have you failed to do what was normally expected of you because of drinking? 6. During the past year, how often have you needed a drink in the morning to get yourself going after a heavy drinking session? 7. During the past year, how often have you had a feeling of guilt or remorse after drinking? 8. During the past year, have you been unable to remember what happened the night before because you had been drinking? 9. Have you or someone else been injured as a result of your drinking? and problematic drinking behavior was defined as AUDIT-Score > 5 in women or >8 in men^14,15^.

### Laboratory measurement

The laboratory parameters were analyzed by a certified laboratory (Labor 28 GmbH and Labor Berlin GmbH, both Berlin, Germany). Blood samples were drawn from all participants after >8 hours fasting and kept at 4–8 °C until analysis on the same day. An oral glucose tolerance test (OGTT) was performed according to the WHO-guidelines in subjects without self-reported diabetes (T2D)^19^. Glucose levels (fasting and 2-hours post load) were measured using photometric methods and insulin levels were determined by an electrochemiluminescence immunoassay (Elecsys® Insulin, Cobas/Roche). HbA1c was measured using high-performance chromatography (VARIANT II TURBO HbA1c Kit – 2.0, Bio-Rad). Thyroid-stimulating hormone (TSH) was measured by electrochemiluminescence immunoassay (ECLIA). C-reactive protein (CRP) was determined in serum samples by means of an immunoassay.

To estimate dietary intake of total energy participants completed a validated, self-administered 146-item food frequency questionnaire (European Prospective Investigation into Cancer and Nutrition)^20^.

### Dual-energy X-ray absorptiometry (DXA)

Body composition was assessed with DXA Hologic Discovery Wi (software APEX version 3.0.1). A trained technician performed the DXA measurement protocol. Appendicular lean mass (ALM) in kilograms was calculated as the sum of the non-bone lean mass in arms and legs.

### Co-variables

Standardized questions were used to ask for current or former smoking status (yes/no) and antidiabetic medication (no therapy/oral antidiabetic medication/insulin treatment. We used one item from the Rapid Assessment of Physical Activity (RAPA) questionnaire “I rarely or never do any physical activities” (yes/no) to assess physical inactivity. Maximal isometric hand grip strength was measured on the left-hand side using a Smedley Dynamometer (Scandidact, Denmark). The geriatric depression scale (GDS) was used as a psychometric measure of depressive symptoms^21^. A morbidity index largely based on the categories of the Charlson index was computed, which is a weighted sum of moderate to severe, mostly chronic physical illnesses, including cardiovascular (e.g., congestive heart failure), cancer (e.g., lymphoma), and metabolic diseases (e.g., diabetes mellitus)^22,23^.

### Statistical analysis

The statistical analysis was carried out using the software package SPSS 24 for Windows. Table 1 displays the results of Two-way ANOVA analysis according to T2D-status and sex. Group comparisons (parameters of the AUDIT questionnaire with sex or T2D status) were computed using Chi2-Test. The results of different linear regression models – with grip strength and ALM as dependent variable – were calculated to assess the association of problematic drinking behavior (yes/no) with dependent variables, next to potential co-variables (Tables 2 and 3 and Supplementary Tables 1 and 2). Models were separately calculated according to T2D status and sex and adjusted for an increasing number of co-variables (fully adjusted model 3: adjustment for age, trunk fat mass, HbA1c, antidiabetic medication, TSH, CRP, testosterone, physical activity level, depression (GDS-score), morbidities, smoking status and total energy intake/day. To rule out that two or more of the used factors correlate strongly with each other, multicollinearity test was performed and VIF values above 5 were considered “too high”. To secure against problematic distribution characteristics and/or outliers and to test stability and reliability of our models, we performed bootstrap analysis, which is displayed in Supplementary Tables 3 and 4.

**Table 1:** Clinical characteristics of the BASE-II study population according to T2D and sex.

	Men (n = 714)		Women (n = 737)		p-value^a^	p-value^b^
	no T2D (n = 598)	T2D (n = 116)	no T2D (n = 672)	T2D = (65)		
Age [years]	69 ± 4	68 ± 4	68 ± 4	68 ± 3	0.105	0.368
BMI [kg/m²]	26.9 ± 3.3	29.5 ± 4.3	26.1 ± 4.4	30.2 ± 5.1	0.992	<0.001
Trunk fat [Kg]	13.7 ± 4.2	16.7 ± 4.9	13.4 ± 4.8	17.4 ± 4.6	0.692	<0.001
ALM [kg]	25.5 ± 3.0	26.0 ± 3.5	17.1 ± 2.4	17.9 ± 2.6	<0.001	0.003
Grip strength [kg]	42 ± 6.9	41.8 ± 7.7	26.5 ± 4.9	26.4 ± 5.8	<0.001	0.795
**Alcohol intake**						
Problematic drinking (Audit > 5 in women or >8 in men) [n; %]	62 (10.4)	24 (20.7)	69 (10.3)	7 (10.8)	0.053	0.037
Alcohol Frequency (alcohol intake/week) [n; %]						
Monthly or less	74 (12.4)	19 (16.4)	167 (24.9)	26 (40.0)	<0.001	0.129
2 to 4 times a month	112 (18.7)	23 (19.8)	218 (32.4)	12 (18.5)		
2 to 3 times a week	170 (28.4)	26 (22.4)	148 (22.0)	15 (23.1)		
4 or more times a week	242 (40.5)	48 (41.4)	139 (20.7)	12 (18.5)		
Alcohol quantity (drinks/day) [n; %]						
1 to 2	434 (72.6)	75 (64.7)	571 (85.0)	56 (86.2)	<0.001	0.577
3 to 4	137 (22.9)	38 (32.8)	94 (14.0)	8 (12.3)		
5 to 6	19 (3.2)	1 (0.9)	6 (0.9)	1 (1.5)		
7 to 9	4 (0.7)	2 (1.7)	1 (0.1)	0 (0.0)		
10 or more	4 (0.7)	0 (0.0)	0 (0.0)	0 (0.0)		
ALAT (U/L)	23 ± 10	31 ± 16	19 ± 8	24 ± 10	<0.001	<0.001
ASAT (U/L)	25 ± 6	28 ± 10	24 ± 6	25 ± 7	<0.001	<0.001
Gamma - GT (U/L)	34 ± 31	50 ± 71	23 ± 23	30 ± 18	<0.001	<0.001
HbA1c (%)	5.5 ± 0.4	6.6 ± 0.9	5.5 ± 0.3	6.4 ± 0.8	0.019	<0.001
TSH (mU/L)	2.1 ± 2.8	2.2 ± 3.0	2.3 ± 4.3	2.3 ± 3.5	0.544	0.844
CRP (mg/L)	1.8 ± 2.9	± 2.1 ± 2.2	2.1 ± 3.5	± 3 ± 2.9	0.018	0.025
CK (U/L)	123 ± 69	136 ± 96	101 ± 55	111 ± 61	<0.001	0.037
Testosterone (ng/mL)	4.8 ± 1.9	3.9 ± 1.8	0.2 ± 0.4	0.2 ± 0.1	<0.001	<0.001
Total energy intake (kcal/day)	2554.9 ± 702.4	2476.5 ± 756.8	1965.6 ± 546.7	1977.4 ± 638.6	<0.001	0.559

Data are either mean ± SD or number (%). BMI = Body Mass Index; Trunk fat = trunk fat mass by DEXA; ALM = appendicular lean mass by DEXA; ALAT = Alanin-Aminotransferase; ASAT = Aspartat-Aminotransferase; CK = creatinine kinase; CRP = C-reactive protein; T2D = Diabetes mellitus type II.

^a^Statistical significance by sex.

^b^Statistical significance by T2D-status.

**Table 2:** Association between grip strength and problematic drinking behavior in men.

Model	no T2D (n = 598)					p^b^	Model	T2D (n = 116)					p^b^
	Unstandardised coefficients		Model characteristics					Unstandardised coefficients		R^2^	Partial Eta Squared	Observed^a^ power	
	B	SE	R^2^	Partial Eta Squared	Observed^a^ power			B	SE				
1	−0.368	0.956	0.049	<0.001	0.067	0.701	1	−5.242	1.981	0.083	0.074	0.745	0.010
2	0.242	1.155	0.100	<0.001	0.055	0.834	2	−6.843	2.719	0.221	0.131	0.691	0.016
3	0.270	1.163	0.103	<0.001	0.056	0.817	3	−8.115	3.298	0.222	0.134	0.670	0.018

Tests of Between-Subjects Effects with grip strength as dependent variable and problematic drinking behaviors as independent variable; B = Beta coefficient; SE = standard error; T2D = Type II Diabetes.

^a^Computed using alpha = 0.05.

^b^P for trend from linear regression models.

Model 1: Age, trunk fat mass, HbA1c, antidiabetic medication.

Model 2: Model 1 + TSH, CRP, Testosterone, physical activity level (RAPA), depression (GDS-score), morbidities, Smoking status.

Model 3: Model 2 + total energy intake/day.

**Table 3:** Association between muscle mass (ALM) and problematic drinking behavior in men.

Model	no T2D (n = 598)					p^b^	Model	T2D (n = 116)					p^b^
	Unstandardised coefficients		Model characteristics					Unstandardised coefficients			Partial Eta Squared	Observed^a^ power	
	B	SE	R^2^	Partial Eta Squared	Observed^a^ power			B	SE	R^2^			
1	0.434	0.396	0.098	0.002	0.194	0.274	1	−1.748	0.786	0.254	0.049	0.596	0.028
2	0.650	0.463	0.125	0.006	0.289	0.161	2	−2.723	1.063	0.474	0.125	0.708	0.014
3	0.716	0.476	0.124	0.008	0.322	0.134	3	−3.661	1.251	0.481	0.166	0.816	0.005

Tests of Between-Subjects Effects with grip strength as dependent variable and problematic drinking behaviors as independent variable; B = Beta coefficient; SE = standard error; T2D = Type II Diabetes.

^a^Computed using alpha = 0.05.

^b^P for trend from linear regression models.

Model 1: Age, trunk fat mass, HbA1c, antidiabetic medication.

Model 2: Model 1 + TSH, CRP, Testosterone, physical activity level (RAPA), depression (GDS-score), morbidities, Smoking status.

Model 3: Model 2 + total energy intake/day.

### Statement of informed consent

Informed consent was obtained from all patients for being included in the study.

## Results

Complete cross-sectional data on drinking behavior and T2D were available for 1451 BASE-II participants. T2D was prevalent in 12.5% of the study population and 11.2% of the whole sample reported problematic drinking behavior (AUDIT-Score > 5 in women or >8 in men). In 20.7% of male subjects with T2D and in 10.8% of women with T2D problematic drinking behavior was evident. The following results on two-way ANOVA tests according to sex and T2D. Women had lower BMI, ALM, grip strength, HOMA-IR and transaminases (GOT, GPT and GGT, p < 0.001) compared to men whereas men had more frequently T2D (16.2% in men and 8.8% in women) and received antidiabetic medication more often (p < 0.001). Moreover, women reported less frequently to be physically inactive (p = 0.017). Although overall men reported a higher frequency and more servings of drinks per day (p < 0.001), we found no significant difference regarding problematic drinking behaviors comparing men (12%) and women (10.3%). Indeed, more than six servings of drinks/day and concerns regarding alcohol consumption were reported in men more frequently (p < 0.001) and women reported having problems to stop drinking once they had started less frequently (p > 0.077) as well as having suffered injuries (or having injured someone else) because of their drinking behaviors (p = 0.003). Other parameters of the AUDIT questionnaire were comparably distributed in men and women.

We also performed two-way ANOVA-tests according to sex and problematic drinking behaviors. Participants with problematic drinking behavior had higher levels of GGT (p < 0.001), GOT (p = 0.004) and GPT (p < 0.001), higher trunk fat mass (p = 0.062) and were of younger mean age (p < 0.001). Remarkably, men had a higher mean GDS-score (p = 0.015) and women had a higher total energy-intake (p < 0.001) if they reported problematic drinking behavior.

Table 1 shows the distribution of clinical characteristics according to T2D in men and women. We found that male subjects with T2D reported problematic drinking behavior more frequently and were less frequently physically active compared to non-diabetic men. Subjects with T2D had significantly higher BMI, trunk fat mass, CRP, GPT, GGT and HbA1c levels compared to non-diabetics, independent of sex. ALM (unadjusted for weight or height) was significantly higher in subjects with diabetes compared to non-diabetics. Women with T2D reported to drink alcohol less frequently (p = 0.028) but in a comparable quantity if they consumed alcohol. Diabetic men reported concerns about alcohol consumption more frequently (p = 0.07). Other parameters of the AUDIT questionnaire were comparably distributed in subjects with and without diabetes, independent of sex.

In order to assess the association of problematic drinking behavior with muscle mass (ALM) and muscle function (grip strength) we calculated linear regression models stepwise adjusted for an increasing number of covariates (separated models for subjects with T2D and without T2D and separated for sex). Models were adjusted for potential confounders associated with low muscle mass and alcohol consumption (age, BMI, smoking status, morbidities, HbA1c, CRP, TSH, physical activity, testosterone, GDS-score, nutrition and antidiabetic medication). During the preparation of the models, a test for multicollinearity was performed and a VIF < 5 was considered acceptable. As summarized in Tables 2 and 3, in adjusted models problematic drinking behaviors were associated with lower muscle mass (B: −3.7, SE: 1.3, R^2^: 0.481, partial eta square: 0.166, observed power: 0.816, p-value = 0.005) and grip strength (B: −8.1, SE: 3.3, R^2^: 0.222, partial eta square: 0.134, observed power: 0.670, p-value = 0.018) in old diabetic men. These associations were not evident in women and subjects without T2D. Notably, model results in men without T2D and in women had insufficient informative value with respect to R^2^, partial eta squared and observed power (see Tables 2 and 3, as well as Supplementary Tables 1 and 2). To secure against problematic distribution characteristics and/or outliers and to test stability and reliability of our models, we performed bootstrap analysis, which is displayed in Supplementary Tables 3 and 4. Results remained stable with respect to the ALM-problematic drinking behavior association but only a trend could be observed with respect to the grip strength-problematic drinking behavior association in old T2D men. Finally, we recalculated model 3 with the AUDIT-score as a continuous variable instead of a dichotomous variable. Results remained comparable with a statistically significant association between AUDIT-score with ALM (B: −0.35, SE: 0.154, R^2^: 0.442, p-value = 0.030) and a trend towards an association between grip strength and AUDIT-score (B: −0.71, SE: 0.40, R^2^: 0.168, p-value = 0.084), solely in old men with T2D.

## Discussion

In the present analysis of BASE-II data we found that problematic drinking behavior was negatively associated with muscle mass (ALM) and muscle function (grip strength) in older men with diabetes. However, there was no statistically significant difference regarding handgrip strength and muscle mass in non-diabetic subjects and women with diabetes. In addition, men with T2D reported problematic drinking behaviors and low physical activity more frequently, and had higher levels of GGT, when compared to non-diabetic men.

Loss of muscle mass has not only been recognized in subjects with T2D but also in acute or chronic alcohol consumption^24,25^. There are several mechanisms through which T2D and muscle mass are linked. Muscle tissue is involved in glucose/insulin metabolism and particularly the PI3K/Akt pathway plays a special role here, as an insufficient activation of this pathway is seen in T2D and accelerates breakdown of skeletal muscle^26^. Additionally, diabetic subjects are at increased risk for hypoglycemic conditions, particularly when alcohol is consumed^27^. Alcohol causes delayed hypoglycemia, which is particularly problematic in diabetics who inject insulin or take certain oral antidiabetic medications, especially if application of antidiabetic drugs is not well-controlled^27,28^. There are various explanations for the possible causes of hypoglycemia. Alcohol temporarily increases the pancreatic blood microcirculation and increases late-phase insulin secretion from beta cells^29^. Moreover, alcohol promotes inhibition of hepatic gluconeogenesis, thus hypoglycemic conditions are favored if alcohol is consumed carelessly. Such hypoglycemic conditions are discussed as a potential link between T2D, alcohol consumption and low muscle mass, sarcopenia and frailty^12^. In addition, it has to be considered, that chronic alcohol consumption is associated with low physical activity, smoking habits, morbidities, and an unbalanced diet, which is additionally associated with protein deficiency and subsequently poor muscle mass^30–34^.

There is, moreover, a strong association of T2D with higher fat mass and BMI^35^, which was also true in the cohort studied here. Investigation of the interplay between obesity, insulin resistance and muscle mass/muscle strength is challenging, especially regarding the definition of sarcopenia. We already showed that a correction of height and weight is essential to calculate ALM in the context of metabolic impairment as seen in T2D^36^. Taking this into account, results regarding associations between ALM and T2D (Table 1) have to be interpreted with caution. In obese individuals, insulin resistance promotes muscle catabolism, because insulin is a powerful anabolic signal^37^. Elevated concentrations of IL-6 and CRP are often detected in sarcopenia and sarcopenic obesity (SO)^38^. SO is associated with metabolic syndrome and low-grade inflammation. Moreover, central obesity affects inflammation and may therefore impair muscle function and promote SO^39^. Knowing the influence of obesity on a potential association between drinking behaviors and muscle mass in T2D, we decided to correct regression models for trunk fat mass measured by DEXA. Nevertheless, there is still discussion on how obesity may interplay with the development of sarcopenia, especially with respect to different life spans^40^.

Although we can partly assume the pathophysiology of the relationship between problematic drinking behaviors in T2D and loss of muscle mass and muscle function, the question arises why these results in the current analysis were solely evident in men. In the current analysis, on average men with T2D reported more servings of drinks per day, and drinking on more days in a week/month when compared to non-diabetic men. In addition, more than six drinks/day and concerns regarding alcohol consumption were reported more often from diabetic men. Women with T2D, however, reported to drink alcohol less frequently, whereas other parameters of the AUDIT-questionnaire were equally distributed in women with and without T2D. This might be one reason why we found that only in diabetic men problematic drinking behavior was associated with lower muscle mass and grip strength, as women may be better informed about negative consequences of alcohol in T2D or drink with more caution when diagnosed with T2D^41^. Moreover, low muscle mass and muscle strength was only seen in men with diabetes, who reported low physical activity more often and problematic drinking behavior more frequently than women. It seems that especially in this subgroup, alcohol consumption may accelerate loss of muscle mass and muscle function as problematic drinking behavior was independently associated with low ALM and grip strength (independent of i.e. physical activity, BMI or antidiabetic medication). Repeated hypoglycemia, as might more frequently occur in this subgroup potentially contributes to the loss of muscle mass/function. Notably, diabetic men received more often antidiabetic medication than women. The use of antidiabetic medication may aggravate the risk/frequency of hypoglycemic states in men. The factors discussed above might explain, at least in part, our observation of an association between problematic drinking behavior with muscle mass/muscle function and its restriction to men. Altered fat distribution or lifestyle parameters might complementary contribute to this finding, as well as possible differences in T2D severity between men and women.

Our results are subject to limitations. First, the current dataset is based on cross-sectional information, thus conclusions on causality cannot be drawn. Although we discuss various potential mechanisms which could drive the results found in the current analysis, no measures of i.e. hypoglycemia over time or adherence to medication were available. With respect to our statistical approach and due to the multiple tests performed – although i.e. test for multicollinearity and bootstrap analysis was performed - there is a certain probability of incidental findings. We have to point out, that the aim of the analysis, however, was to further substantiate a hypothesis, which needs to be further tested in a longitudinal setting. Notably, model results in men without T2D and in women had insufficient informative value with respect to R^2^, partial eta squared and observed power (see Tables 2 and 3, as well as Supplementary Tables 1 and 2). Model stability, R^2^ and observed power of the main results model (model 3, men with T2D) were good and stable, with respect to the ALM-problematic drinking behavior association, however only a trend could be observed with respect to the grip strength-problematic drinking behavior association in old T2D men. Although we calculated with a dataset of 1451 BASE-II subjects, it has to be mentioned that in some calculations a small number (i.e. 20.7% of male subjects with T2D and in 10.8% of women with T2D reported problematic drinking behavior) of subjects remained when subgroups were compared.

There are various confounders associated both with drinking behavior and muscle mass/muscle function. The cause-effect relationship cannot be concluded from a cross-sectional design. Thus i.e. physical inactivity or depression might be the reason for problematic drinking behavior or low muscle mass but could also be interpreted as cause of these states. Furthermore, the data on problematic drinking behavior were self-reported (questionnaire) and thus subjective. Information about duration of or amount of alcohol consumption (i.e. g/day of alcohol) is not part auf this questionnaire. The lack of intermediate categories in the framework of a questionnaire means that some categories may be over- or underestimated. The same is true for the questions about physical inactivity and nutrition. Physical inactivity was analyzed solely by one question (participants were asked if they “rarely or never do any physical activities” (yes/no)), not by objective means. This question is based on the RAPA-questionnaire, which has recently been called into question, especially for the use in older persons^42^. Nevertheless, the AUDIT questionnaire and the European Prospective Investigation into Cancer and Nutrition questionnaire are well established instruments to assess problematic drinking behavior and nutritional intake. Finally, the BASE-II cohort is a typical convenience sample. Diseases such as chronic obstructive pulmonary disease (COPD) and coronary heart disease are under-represented, thus the results from this study cohort cannot simply be extrapolated to the general population.

In conclusion, we found a negative association between problematic drinking behavior and muscle mass/muscle function in old diabetic men. These subjects might be at increased risk for loss of muscle mass and early functional decline, as muscle function and muscle mass may be reduced due to problematic alcohol consumption (i.e. as a result of hypoglycemic conditions, because of higher sensitivity of muscle mass to alcohol or due to higher alcohol intake in these subjects). However, the cross-sectional design of the current analysis does not allow to draw conclusions on causalities. Nevertheless, our results indicate the need for large and longitudinal studies to confirm these findings in old subjects with T2D and to clarify, whether problematic drinking behavior might favor loss of muscle mass and muscle function. These subjects might benefit from more intensive information about negative consequences of alcohol consumption in T2D and should be educated accordingly to prevent early functional decline.

## Supplementary information

Supplement Tables

## Data Availability

Due to concerns for participant privacy, data are available only upon request. External scientists may apply to the Steering Committee of BASE-II for data access. Please contact Katrin Schaar, scientific coordinator, at schaar@mpib-berlin.mpg.de.
