# Supplementary material for: Problematic drinking in the old and its association with muscle mass and muscle function in type II diabetes
Source: Sci Rep. 2019 Aug 19;9:12005. doi: 10.1038/s41598-019-47787-0 (PMC6700201; doi:10.1038/s41598-019-47787-0)
Supplement: Supplementary file 1 — Supplement Tables [file 41598_2019_47787_MOESM1_ESM.pdf]

Problematic drinking in the old and its association with muscle mass and muscle function in type II diabetes

Nikolaus Buchmann, Dominik Spira, Maximilian König, Kristina Norman, Ilja Demuth and Elisabeth Steinhagen-Thiessen

Supplement table 1: Association between grip strength and problematic drinking behavior in women

	**no T2D (n = 672)**								**T2D (n = 65)**					
Model	Unstandardised coefficients		Model characteristics					Model	Unstandardised coefficients					
	B	SE	R^2	Partial Eta Squared	Observed[a] power	p[b]			B	SE	R^2	Partial Eta Squared	Observed[a] power	p[b]
1	-0.040	0.678	0.012	<0.001	0.050	0.953		1	2.702	2.430	0.216	0.024	0.194	0.271
2	-1.181	0.882	0.069	0.006	0.267	0.181		2	5.068	4.891	0.428	0.067	0.163	0.317
3	-0.821	0.916	0.088	0.003	0.145	0.371		3	9.712	4.697	0.584	0.263	0.477	0.061

B = Beta coefficient; SE = standard error; T2D = Type II Diabetes

a) Computed using alpha = 0.05
b) P for trend from linear regression models

Model 1: Age, trunk fat mass, HbA1c, antidiabetic medication
Model 2: Model 1 + TSH, CRP, Testosterone, physical activity level (RAPA), depression (GDS-score), morbidities, Smoking status
Model 3: Model 2 + total energy intake/day

Supplement table 2: Association between ALM and problematic drinking behavior in women

	no T2D (n = 672)								T2D (n = 65)					
Model	Unstandardised coefficients		Model characteristics					Model	Unstandardised coefficients					
	B	SE	R^2	Partial Eta Squared	Observed[a] power	p[b]			B	SE	R^2	Partial Eta Squared	Observed[a] power	p[b]
1	0.286	0.295	0.167	0.002	0.162	0.333		1	0.676	1.074	0.197	0.007	0.095	0.532
2	0.326	0.399	0.231	0.002	0.129	0.415		2	0.162	2.178	0.343	0.000	0.051	0.941
3	0.245	0.416	0.235	0.001	0.090	0.557		3	0.626	2.084	0.389	0.006	0.059	0.768

B = Beta coefficient; SE = standard error; T2D = Type II Diabetes

a) Computed using alpha = 0.05
b) P for trend from linear regression models

Model 1: Age, trunk fat mass, HbA1c, antidiabetic medication
Model 2: Model 1 + TSH, CRP, Testosterone, physical activity level (RAPA), depression (GDS-score), morbidities, Smoking status
Model 3: Model 2 + total energy intake/day

Supplement table 3: Results of bootstrap for coefficients of linear regression model 3 with respect to the association between ALM and problematic drinking behavior

		Unstandardised coefficients			BCa 95% confidence interval	
		B	SE	p-value	Lower bound	Upper bound
Men	No T2D **(n = 598)**	0.716	0.512	0.157	-0.268	1.690
	T2D **(n = 116)**	-3.661	1.775	0.037	-7.812	-0.269
women	No T2D **(n = 672)**	0.245	0.482	0.582	-0.787	1.391
	T2D **(n = 65)**	0.626	5.217	0.883	-10.065	10.997

Based on 1000 samples

Supplement table 4: Results of bootstrap for coefficients of linear regression model 3 with respect to the association between grip strength and problematic drinking behavior

		Unstandardised coefficients			BCa 95% confidence interval	
		B	SE	p-value	Lower bound	Upper bound
Men	No T2D **(n = 598)**	0.270	1.061	0.797	-1.925	2.373
	T2D **(n = 116)**	-8.115	4.692	0.075	-18.429	2.700
women	No T2D **(n = 672)**	-0.821	0.986	0.425	-2.561	0.769
	T2D **(n = 65)**	9.712	17.337	0.431	-22.426	36.086

Based on 1000 samples
